# Rhabdomyosarcoma in children: Retrospective analysis from a single tertiary care center in Saudi Arabia

**DOI:** 10.1002/cnr2.1683

**Published:** 2022-08-09

**Authors:** Samer N. Markiz, Safia Khan, Zainab B. Wagley, Mohammed K. Viqaruddin, Yasser M. Khafaga, Ibrahim A. AlFawaz, Awatif E. AlAnazi, Amani AlKofide, Hatem A. Khoja, Afshan A. Ali

**Affiliations:** ^1^ Department of Pediatric Hematology/Oncology King Faisal Specialist Hospital & Research Centre Riyadh Saudi Arabia; ^2^ Department of Pediatrics Central Park Teaching Hospital Lahore Pakistan; ^3^ AlFaisal University School of Medicine Riyadh Saudi Arabia; ^4^ Department of Radiation Oncology King Faisal Specialist Hospital & Research Centre Riyadh Saudi Arabia; ^5^ Department of Pathology & Laboratory Medicine King Faisal Specialist Hospital & Research Centre Riyadh Saudi Arabia; ^6^ Department of Pediatrics Oncology King Faisal Specialist Hospital & Research Centre Madinah Saudi Arabia

**Keywords:** children cancer, retrospective study, rhabdomyosarcoma, tertiary care

## Abstract

**Background:**

Rhabdomyosarcoma (RMS) is the most common soft tissue sarcoma in children occurring most commonly in the head and neck region. The treatment involves using a multimodality approach including chemotherapy, surgery, and radiation therapy. Survival for patients with localized disease has improved markedly, but the treatment of advanced disease remains a challenge. We report the clinical characteristics and outcome for patients treated at a tertiary care center in Saudi Arabia.

**Methods:**

Patients aged 0–14 years diagnosed with RMS between 2005 and 2018 were included. Statistical analysis was performed using SPSS software. Kaplan–Meier method was used to calculate overall and event free survival. Cox proportional hazards model was used for multivariate analysis.

**Results:**

One hundred and twenty‐four patients were analyzed. The median age was 5.7 years with male predominance (2.4:1). The most common primary sites were head/neck (30%) and the genitourinary tract (25%). Embryonal RMS was present in 81%; alveolar in 19%. Most patients had intermediate risk disease (60%). The 5‐year overall and event free survivals were 64.3% and 53.3%, respectively. Survival was influenced by primary tumor site, histology, and clinical risk group. Unfavorable primary site, high risk stratification, and poor initial response to therapy predicted a poor outcome.

**Conclusion:**

This study provides an insight on the current management outcomes for our patients with RMS. Cytogenetics and molecular diagnostics need to be incorporated as standard of care in the therapeutic approach of our patients. In addition, there is a need for national collaborative efforts to improve the outcome of RMS in children and adolescents.

**Abbreviations**
RMS: rhabdomyosarcoma
COG: Children Oncology Group
IGRSG: Intergroup Rhabdomyosarcoma Study Group
IRB: Institutional Review Board
TNM: tumor, node, metastasis
VAC: vincristine, dactinomycin, cyclophosphamide
VA: vincristine and dactinomycin
VI: vincristine and irinotecan
VDC: vincristine/doxorubicin/cyclophosphamide
IE: ifosfamide/etoposide
RT: radiation therapy
VMAT: volumetric modulated arc therapy
OAR: organs at risk
CR: complete response
PR: partial response
SD: stable disease
PD: progressive disease
SIOP: International Society of Pediatric Oncology
HR: hazard ratio
OS: overall survival
EFS: event free survival
GCP: good clinical practice
SPSS: statistical package for social sciences
Gy: Gray
IRS: International Rhabdomyosarcoma Study
CTV: clinical target volume
PTV: planning target volume
GTV: gross tumor volume
3D CRT: 3‐dimensional conformal radiation therapy
ERMS: embryonal rhabdomyosarcoma
ARMS: alveolar rhabdomyosarcoma
FISH: fluorescence in situ hybridization
GU: genito‐urinary
HN: head and neck
CI: confidence interval

## 1 INTRODUCTION

Rhabdomyosarcoma (RMS) is a malignant neoplasm originating from soft tissues specifically skeletal muscles. It is the most common soft tissue sarcoma and accounts for approximately 8% of pediatric cancers in patients under the age of 15.^1^ It is also the third most common extracranial solid tumor in the pediatric age group following Neuroblastoma and Wilms tumor.^2^, ^3^ More than two thirds of the cases are diagnosed in the first decade of life.^4^ RMS has a slight predilection for male sex and is more common in the Caucasian population.^2^

While most cases occur sporadically, several genetic syndromes have shown predisposition for the development of RMS, such as Li‐Fraumeni, Beckwith Wiedemann syndrome, Neurofibromatosis type 1, Rubinstein‐Tayebi syndrome, Von Recklinghausen disease and Costello syndrome.^5^, ^6^, ^7^, ^8^ In addition, some associated environmental factors include intrauterine exposure to X‐rays and parental recreational drug use.^9^, ^10^

RMS can occur in any soft tissue in the body, but the most common sites are the head/neck (35%–40%), followed by genitourinary tract (20%–25%), and extremities (15%–20%).^11^ Typically, the functions of nearby organs are not affected, and the lesion appears as a lumpy mass. Histologically, RMS is classified into three major subtypes: embryonal, alveolar, and pleomorphic.^12^ Embryonal rhabdomyosarcoma (ERMS) is more common in younger children while the other subtypes are seen more frequently at an older age.^13^ Common cytogenetic changes seen in RMS include translocations 2q35,13q14 or 1p36,13q14 and occur in about 80% of alveolar rhabdomyosarcoma (ARMS). Embryonal RMS, on the other hand, exhibits some non‐specific chromosomal gains or losses.^14^

The treatment of RMS involves a multimodality approach which includes chemotherapy, surgery and/or radiation therapy (RT).^15^ Chemotherapy plays a major role in the treatment of patients with RMS.^16^ Commonly used and effective agents include vincristine, dactinomycin and cyclophosphamide. Other agents used with higher risk or advanced disease are etoposide, ifosfamide, cisplatin, irinotecan, temozolomide and vinorelbine. Surgical resection is done upfront whenever possible and when the disease is localized, while radiation therapy is often used for patients with unresectable primary tumors, residual microscopic disease after surgery, or for those having more advanced disease where surgery is not an option. The management strategy is determined by several factors including the primary tumor site, clinical group (based on the Intergroup Rhabdomyosarcoma Study Group (IGRSG), TNM stage (tumor, node, metastasis) and histology.^17^, ^18^

With advances in the treatment approach over time, the survival of patients with RMS has improved markedly particularly in patients under the age of 10.^19^ Several factors that influence the prognosis of RMS include age at diagnosis, disease stage, histology, and primary tumor site. Younger age, localized disease, embryonal histology, orbital and genitourinary sites are associated with better prognosis.^20^ The 5‐year overall survival (OS) is reported to be more than 90% for pediatric RMS patients with localized disease treated with multi‐agent chemotherapy, surgery, and radiation. However, the survival for metastatic disease remains dismal and is reported to be less than 30%.^15^, ^18^ Additional prognostic indicators such as the presence or absence of FOXO1 fusion gene in alveolar type of RMS, the extent of disease, and even circulating tumor DNA have been used to predict outcome.^21^ Studies from the Children's Oncology Group (COG) demonstrated that FOXO1 fusion status is the second most important prognostic factor for predicting survival outcome after metastatic disease thus justifying its incorporation in risk stratification.^22^, ^23^ The management of disease recurrence remains a challenge.

In this study, we performed a retrospective review of pediatric RMS cases that were treated at our center between January 1, 2005, and December 31, 2018. The clinical features, pathological subtypes, and molecular studies were reported. The treatment approaches and survival outcomes were analyzed and compared to what is being reported in the literature.

## 2 MATERIALS AND METHODS

The proposal was reviewed and approved by the Institutional Review Board (IRB) and a waiver of informed consent was granted for this retrospective study. Demographic characteristics, clinical manifestations (including the presenting signs/symptoms, tumor primary site, pathological features, and extent of disease), response to treatment and outcomes were reviewed from patient medical charts both electronic and paper. Data was collected and entered using Redcap (Vanderbilt University), a web‐based data management system. Patient confidentiality was maintained as per the Good Clinical Practice (GCP) guidelines.

### 2.1 Statistical analysis

Baseline characteristics and demographic data were described using frequencies and percentages for categorical values. Kaplan–Meier method was used to calculate 5 and 10‐year overall survival (OS) and event‐free survival (EFS). An event was defined as death, relapse, or new malignancy. The effects of demographic, pathologic, clinical and treatment variables on survival rates were tested utilizing the log‐rank test for categorical values. In addition, Cox regression model was introduced to perform multivariate analysis for prognostic factors found to be significant in the univariate analysis, and proportional hazard assumptions were verified on final models by means of Schoenfeld residuals. All tests were two‐sided and *p*‐value of <.05 (log rank) was considered of statistical significance. Statistical Package for Social Sciences (SPSS) software version 20.0 by SPSS Inc. Chicago, IL, was utilized to perform the statistical analysis.

### 2.2 Patient population and demographics

All children aged ≤14 years who were diagnosed with RMS between 2005 and 2018 at King Faisal Specialist Hospital & Research Center, Riyadh were reviewed. Only previously untreated patients (treatment naïve) were included in the review. Children who were referred for continuation of chemotherapy, radiation therapy, and surgery or were off therapy and seen only as follow‐up at our institution were excluded. Of the 144 patients reviewed, a cohort of 124 were eligible for analysis. In all cases, histopathological diagnosis was confirmed by the institutional pathologist before treatment was started. Of the 124 children, 87 were boys and 37 were girls (ratio 2.4:1). All patients were Saudi nationals and categorized as white Caucasian.

### 2.3 Histological and molecular diagnosis

The final diagnosis was based on morphological review of the pathology specimens supported by immuno‐histochemical staining for Desmin, Myogenin and MyoD1. Cytogenetic testing for the FOXO1 fusion gene was reserved only for cases where the diagnosis was questionable. In such cases, a negative FOXO1 fusion test was considered supportive of the diagnosis of embryonal RMS.

### 2.4 Clinical staging and risk stratification

After confirmation of diagnosis, tumor stage was assigned according to the TNM staging system for solid tumors. The patients' clinical risk group was determined based on the current COG risk stratification for RMS as defined by the IGRSG which incorporates the clinical stage (TNM), clinical group (based on the extent of disease) and histological subtype. All patients were categorized as low‐risk, intermediate‐risk or high‐risk.

### 2.5 Treatment

Treatment approach for RMS has remained consistent over the study period with most children undergoing an initial diagnostic biopsy (*n* = 107), followed by neoadjuvant chemotherapy, local control with surgery where feasible and plus or minus radiation therapy. Some variability in chemotherapy was noted due the shift in the standards of care for RMS including dose reduction of agents such as cyclophosphamide as well as a decrease in the total number of cyclophosphamide cycles and the incorporation of irinotecan for intermediate and high‐risk patients over the last 14 years. Standard of care arms of COG Clinical Trials were incorporated in the clinical management of RMS where low‐risk patients receive 4 cycles of vincristine/dactinomycin/cyclophosphamide (VAC) and 12 cycles of vincristine/dactinomycin (VA); intermediate‐risk patients receive up to 7 cycles of VAC and vincristine/irinotecan (VI) each. High‐risk patients are treated with a regimen containing VI, vincristine/doxorubicin/cyclophosphamide (VDC), ifosfamide/etoposide (IE), and VAC. Prior to 2011, patients received therapy with a VAC containing regimen which served as the backbone for low‐risk and intermediate‐risk group patients while high‐risk groups received VAC alternating with cisplatin/ifosfamide/etoposide. Gross and microscopic surgical margins were used to define the extent of surgical resection. Complete resection was defined as microscopically free margins on histologic evaluation.

### 2.6 Radiation therapy

Ninety‐two patients were treated with RT, either alone (67) or combined with surgery (RT+ Surgery). Sixty‐eight patients with group 3 disease were treated with RT only for local control. The RT dose ranged from 45 to 59.4 Gy, at 1.8Gy fractional dose. Cases with orbital RMS received 45 Gy. For all other group 3 sites (non‐orbit), RT dose ranged from 50.4 to 59.4 Gy, with reduction of RT volume (boost) after 36–41.4 Gy.

Twenty‐four patients had combined surgery and RT for local control. Positive surgical margins ± nodal involvement were the main indications for postoperative RT. In this group of patients, the RT dose ranged from 41.4 to 45 Gy.

Volumetric modulated arc therapy (VMAT) technique was used in axial, pelvic, head and neck, and para‐meningeal sites, whereas most of the extremities RMS were treated with three‐dimensional conformal RT (3D CRT). Gross tumor volume (GTV), Clinical target volume (CTV), Planning target volume (PTV) and organs at risk (OAR) were contoured according to IRS/COG guidelines. The timing of RT varied, with most of the patients starting RT on week 12 chemotherapy (range week 4–16).

## 3 RESULTS

**FIGURE 1:**
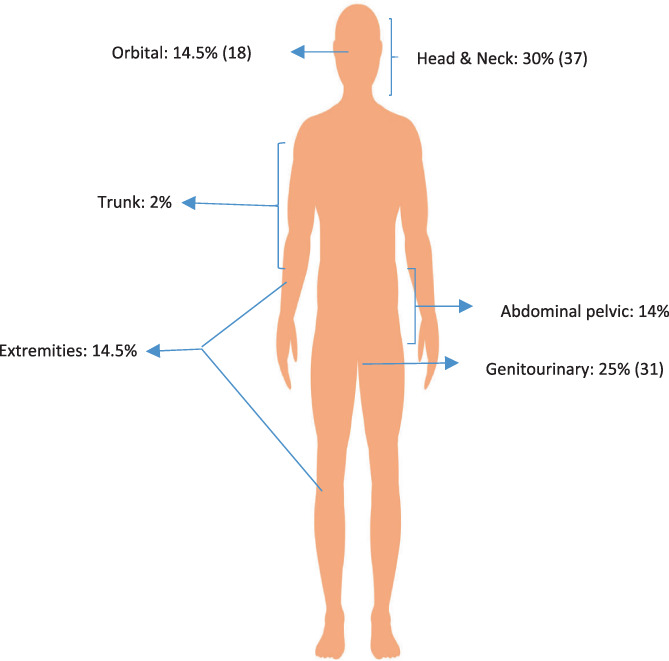
Frequency for RMS site of origin

Of the total 144 pediatric patients identified with RMS during the period 2005–2018, 124 were eligible for analysis. Patient demographics and tumor characteristics are summarized in Table 1. The median age was 5.7 years and ranged between 0.7 years (8 months) and 14 years. Most of the patients were diagnosed at or before the age of 10 years (*n* = 103, 83%). About % of the patients were males, with a male to female ratio of 2.4. An asymptomatic mass was the most common presenting symptom in 55% of the cases. The most frequently affected sites were head and neck (30%), followed by genitourinary tract (25%), Orbit (14.5%) and Extremities (14.5%) (Figure 1). Most of the patients were diagnosed with Embryonal subtype (*n* = 100, 81%), while alveolar histology was seen in 24 patients (19%). Cytogenetic testing for the FOXO1 fusion genes was done in only 44 patients using fluorescent in situ hybridization (FISH). The test was positive is 8 of the 44 patients or 18.2%. Other chromosomal aberrations were found in 21 patients and included extra copies of 13q in 16 patients; loss of 13q in two cases; 18q loss in one patient, tetrasomy 2 in one patient, and pentasomy 8 in one patient. The clinical risk grouping was unified for patients using clinical stage and pathology into low, intermediate, and high‐risk categories. COG risk stratification for RMS was used to re‐classify patients treated prior to standardization of the center's treatment to COG standard arm therapy. Based on that, the cohort was classified as low risk (17%), intermediate risk (60%), and high risk (23%). Upfront tumor resection was only done in 15% of cases, with the most frequently resected tumors being in the head/neck and genitourinary sites. The remaining 85% of patients had initial biopsy as diagnostic modality. With regards to local control, radiation therapy was the most frequently used modality (57%), followed by combined surgery and radiation therapy (19%), and surgery alone (19%). Local control was not done in 5% of the patients because of disease progression or parental refusal.

**TABLE 1:** Demographics and clinical characteristics

	*n*	% of total
Gender		
Male	87	70
Female	37	30
Age		
Median (yrs)	5.7	‐
Mean (yrs)	5.1	‐
Range (yrs)	0.7–14.9	‐
≤10 yrs	103	83
>10 yrs	21	17
Presenting symptoms		
Masses	67	55
Lymphadenopathy	38	31
Abdominal distension	32	26
Fever	27	24
Proptosis	24	23
Dysuria	23	20
Bone pain	20	17
Headache	17	13
Respiratory difficulty	14	11
Frequent micturation	11	9
Anemia	9	7
Haematuria	8	6
Histopathological subtypes		
Embryonal	100	81
Alveolar	24	19
Tumor site		
Head & neck (HN) non‐parameningeal	25	20
Orbital	18	14.5
Genito‐urinary (GU) non‐bladder prostate	6	5
Head & neck (HN) parameningeal	12	10
Extremities	18	14.5
Abdominal/pelvic	17	14
Genito‐urinary (GU) bladder prostate	25	20
Trunk	3	2
Prognostic sites		
Favorable	49	39.5
Unfavorable	75	60.5
IRS risk group		
Low risk	21	17
Intermediate risk	74	60
High risk	29	23
Staging		
Stage‐1	14	11
Stage‐2	14	11
Stage‐3	67	54
Stage‐4	29	23
Surgery		
No surgery	76	61
Surgery	48	39
Radiation therapy		
No radiation	33	25
Radiation	91	75

**FIGURE 2:**
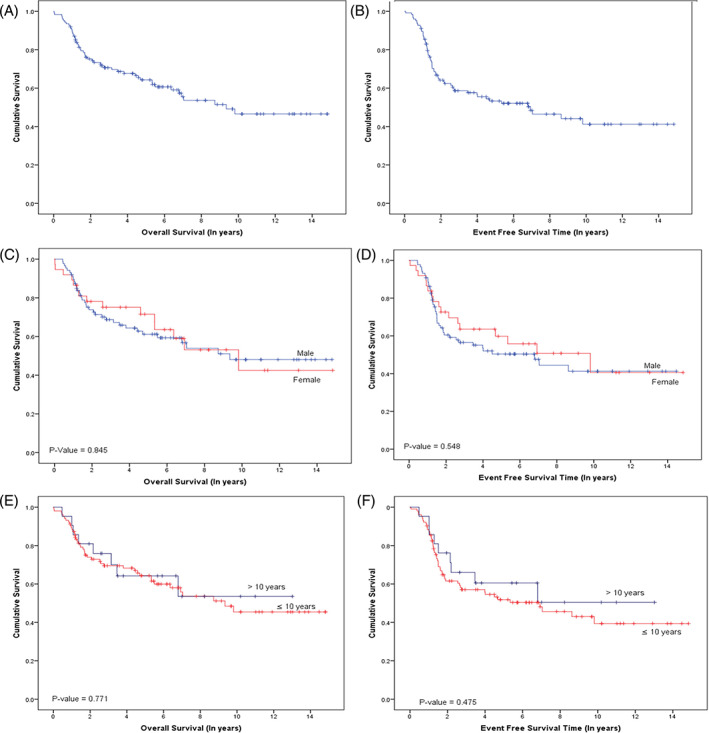
Overall and Event free survival of RMS, retrospective study: (A) Overall survival (OS) of the cohort;(B) Event free survival of the cohort; (C) Overall survival for gender; (D) Event free survival for gender; (E) Overall survival for age group; (F) Event free survival for age group

**FIGURE 3:**
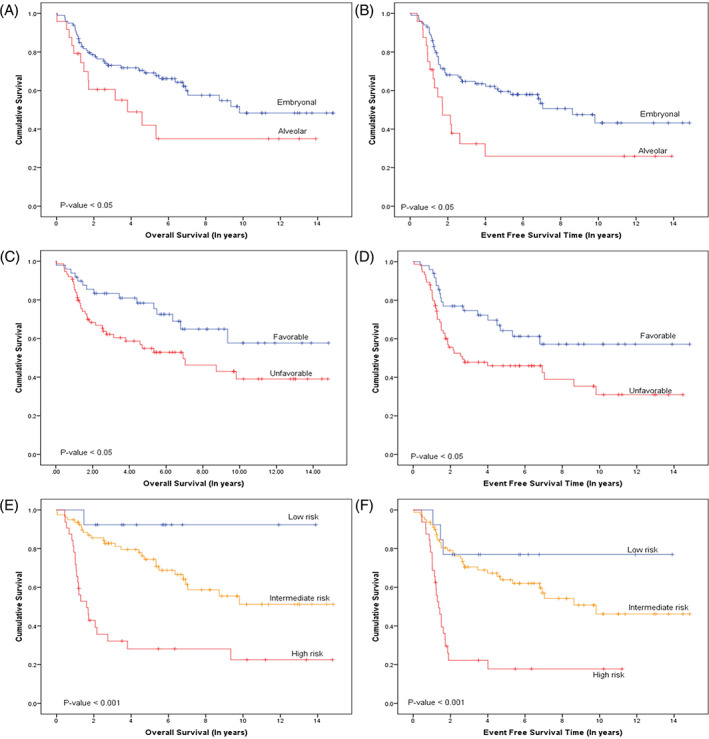
Overall and Event free survival of RMS, retrospective study: (A) Overall survival (OS) of histological sub‐type; (B) Event free survival of histological sub‐type; (C) Overall survival for disease‐site classification: favorable and unfavorable; (D) Event free survival for disease‐site classification: favorable and unfavorable; (E) Overall survival for clinical risk groups: low risk, intermediate risk, high risk; (F) Event free survival for clinical risk groups: low risk, intermediate risk, high risk

Survival data was reported for the whole patient cohort, across all risk groups, with a 5‐year OS of 64.3% and EFS of 53.3% (Figure 2). Tables 2 and 3 summarize the effect of patient demographics and disease characteristics on survival, while age and gender had no impact on survival outcomes (Figure 2), while tumor histology, primary tumor site, and clinical risk group played a significant role. Embryonal histology had a better 5‐year OS (69.1%) and EFS (41.9%) compared to the alveolar subtype (59.5% and 25.9%, respectively), while Analysis by the clinical risk groups revealed the best 5‐year OS in low‐risk patients at 92.3% (*p*‐value <.05) (Figure 3). Primary tumor location to a favorable site was associated with a better 5‐year OS in comparison to unfavorable locations (5‐year OS 78.3% vs. 54.9%, *p*‐value <.05) (Figure 3). Orbital tumors showed the best results (5‐year OS of 88.9%) followed by head and neck (5‐year OS of 66%) (*p*‐value <.05).

**TABLE 2:** Overall Survival (OS): 5 and 10‐years based on Clinical Characteristics and initial response to chemotherapy

	5‐year OS (%)	10‐year OS (%)	*p*‐value
Overall	64.3	46.6	‐
Gender			NS
Male	61.2	48.1	
Female	71.5	42.5	
Age			NS
≤ 10 yrs	64.2	53.5	
> 10 yrs	64.3	45.4	
Histopathological subtypes			<.05
Embryonal	69.1	48.3	
Alveolar	41.9	34.9	
Prognostics sites			<.05
Favorable	78.3	57.7	
Unfavorable	54.9	39.0	
IRS risk group			<.001
Low risk	95.2	95.2	
Intermediate risk	70.7	41.3	
High risk	25.2	25.2	
Local control			NS
Surgery alone	69.9	53.3	
Radiation therapy alone	63.5	48.7	
Surgery + Radiation therapy	75.5	51.8	
Initial response to chemotherapy			<.05
Complete remission (CR)	68.4	68.4	
Partial response (PR)	65.5	40.5	
Stable disease (SD)	61.1	45.8	
Progressive disease (PD)	30.0	15.0	

**TABLE 3:** Event‐Free Survival (EFS): 5 and 10‐ years based on Clinical Characteristics and initial response to chemotherapy

	5‐year EFS (%)	10‐year EFS (%)	*p*‐value
Event‐free survival	53.3	41.2	‐
Gender			NS
Male	50.4	41.2	
Female	59.8	40.6	
Age			NS
≤10 yrs	51.8	39.4	
>10 yrs	60.5	50.4	
Histopathological subtypes			<.05
Embryonal	59.5	43.2	
Alveolar	25.9	25.9	
Prognostics sites			<.05
Favorable	64.2	57.2	
Unfavorable	46.0	31.0	
IRS risk group			<.001
Low risk	85.7	85.7	
Intermediate risk	57.8	37.4	
High risk	17.6	17.6	
Local control			NS
Surgery alone	66.2	48.3	
Radiation therapy alone	48.6	41.7	
Surgery + Radiation therapy	63.0	45.9	
Initial response to chemotherapy			<.05
Complete remission (CR)	61.5	61.5	
Partial response (PR)	50.0	32.7	
Stable disease (SD)	61.6	46.2	
Progressive disease (PD)	20.0	20.0	

**FIGURE 4:**
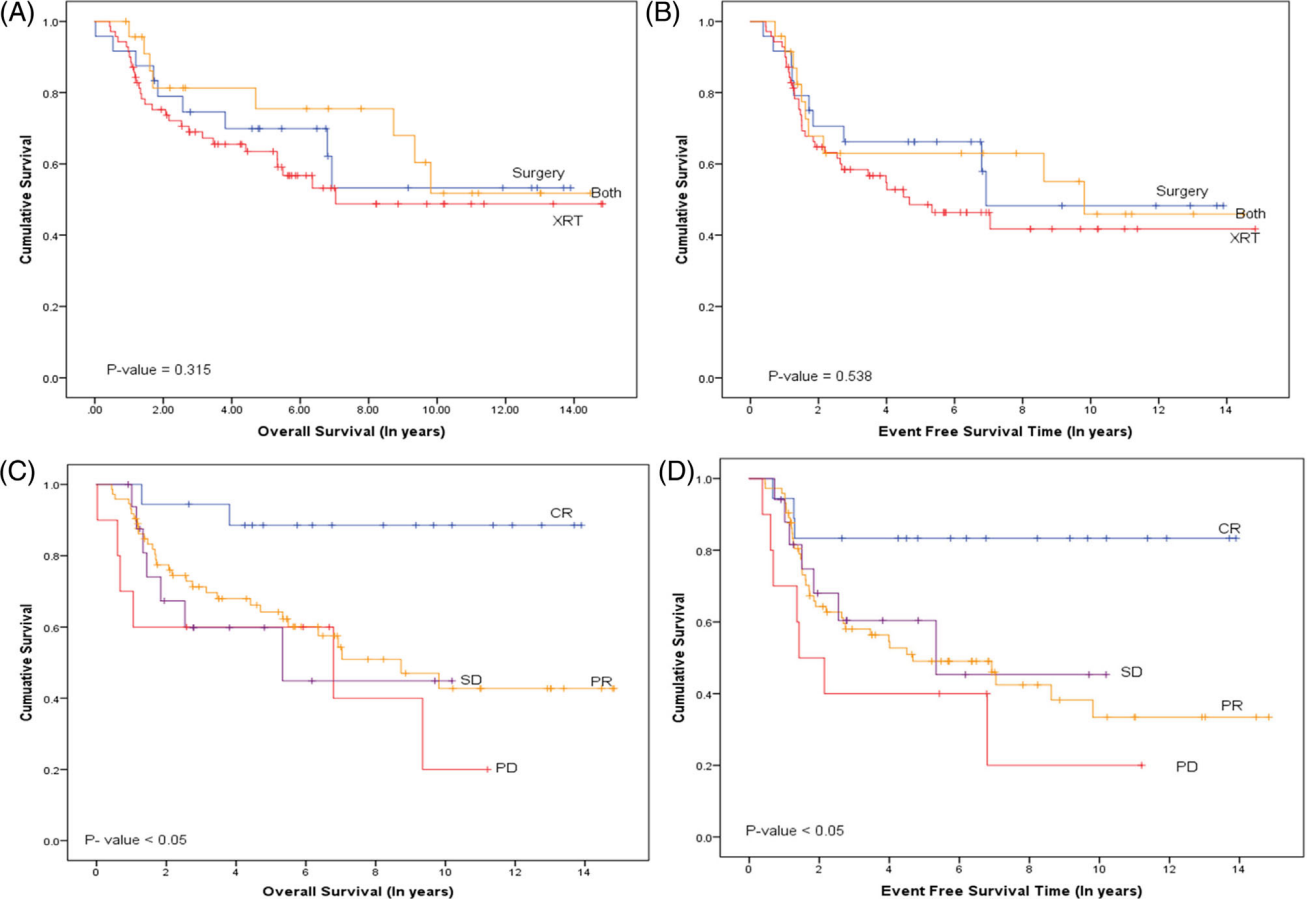
Overall and Event free survival of RMS, retrospective study: (A) Overall survival (OS) of histological sub‐type; (B) Event free survival of histological sub‐type; (C) Overall survival for disease‐site classification: favorable and unfavorable; (D) Event free survival for disease‐site classification: favorable and unfavorable; (E) Overall survival for clinical risk groups: low risk, intermediate risk, high risk; (F) Event free survival for clinical risk groups: low risk, intermediate risk, high risk

We analyzed whether the initial disease response to chemotherapy prior to local control could predict disease outcome. The initial response was determined based on the volumetric measurement for primary tumor site response as used in the COG treatment protocols while the response of metastatic disease was assessed based on the RECIST criteria. The two measures were combined to assign the overall disease response as complete response (CR), partial response (PR), stable disease (SD) or progressive disease (PD). The modality used to make measurements was computed topography (CT) scan. We found that the initial response to chemotherapy had a significant influence on survival outcomes (Figure 4). This influence was not seen after excluding the group of patients who had progressive disease prior to local control. These findings are in agreement with reports from the International Society of Pediatric Oncology (SIOP) and COG.^24^, ^25^

Survival outcomes were not significantly different with respect to the different local control modalities implemented. There was a clear survival advantage of utilizing both radiation and surgery for local control in our patients, but this did not reach statistical significance (Figure 4).

While timing of radiation therapy varied, most of the patients started radiation treatment for local control approximately at week 12 of chemotherapy (range week 4–16). Due to the numbers, outcome analysis comparing the standard versus deferred timing of radiation therapy was not possible.

**FIGURE 5:**
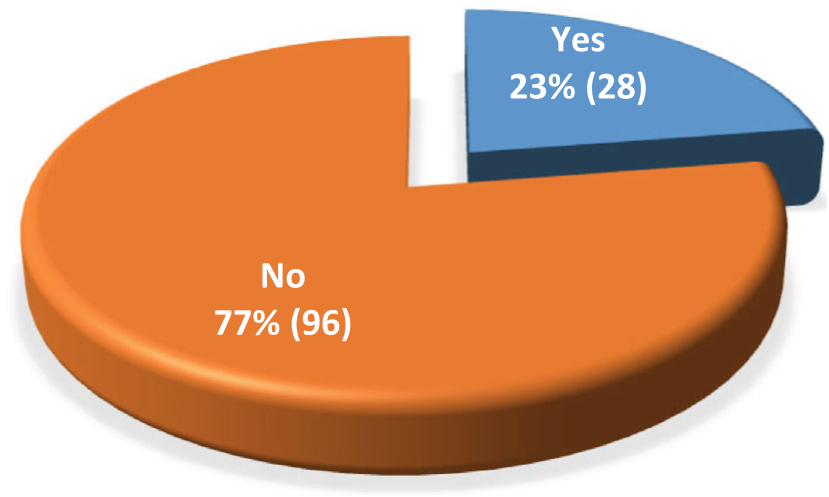
Frequency of RMS relapse, retrospective study

Disease recurrence occurred in 23% (28) of the patients (Figure 5). There were fewer relapses in the embryonal compared to alveolar subtype (21% vs. 50%) (Table 4). Relapse was lowest in the low‐risk group (9.5%) compared to the intermediate‐risk (26%) and high‐risk group (24.1%) (Table 5).

**TABLE 4:** Incidence of relapse by histological subtype

Sub type	Yes
Embryonal	21%(20/97)
Alveolar	50%(8/16)

**TABLE 5:** Incidence of relapse by risk stratification

Risk stratification	Yes
Low risk	9.5% (2/21)
Intermediate risk	26% (19/74)
High risk	24.1% (7/29)

The results of multivariate analysis using the Cox regression model are summarized in Table 6 and indicate that the high‐risk group (HR 2.235; *p*‐value <.001), unfavorable site (HR 1.051; *p*‐value <.05), and poor response to initial chemotherapy (HR 1.388; *p*‐value <.05) were independent predictors of poor outcome in our cohort.

**TABLE 6:** Multivariate analysis

	Hazards Ratio	95% CI		*p*‐value
Gender	0.942	0.515	1.721	.845
Age ≤ 10 yrs	0.420	0.420	1.902	.771
Alveolar type	0.967	0.967	3.427	.063
High risk (Stage 4)	2.235	2.235	5.830	<.001
Unfavorable site	1.051	1.051	3.514	<.05
Poor initial response to chemotherapy	1.388	1.388	1.042	<.05

## 4 DISCUSSION

This retrospective study is a comprehensive analysis of the clinical characteristics and management outcomes for RMS in pediatric patients treated at KFSH&RC‐Riyadh and provides important insight on the local pattern of disease and serves as a tool to benchmark clinical features, treatments, and outcomes against international data. Several key points are addressed below based on the results of the study.

One of the major limitations of this analysis is that the pediatric population in Saudi Arabia is considered up to the age of 14 years, and thus patients between the ages of 14 and 18 are excluded from our analysis as they are treated by the adult services using different treatment plans. While the literature reports that 50%–70% of RMS patients are diagnosed before the age of 10 years, 83% of our patient cohort were younger than 10. This is most likely a reflection of the missing numbers for the age 14–18 years group.^4^, ^15^ Having access to the population between the ages of 14–18 years and incorporating them on the current RMS protocols according to the international standards would provide a more similar demographic area for analysis and benchmarking and would influence the survival data for the whole RMS cohort.

Despite recent improvement in public awareness and the provision of public health campaigns aiming at early detection and prompt treatment of cancer in Saudi Arabia, most of our patients presented with stage 3 and 4 disease (77.4%). This finding might be the result of a delay in seeking medical attention or in referral to a tertiary care facility after the initial symptom onset, which was an asymptomatic mass in around half of our patient population (55%). We observed that more than 50% of the patients treated at our center were referred from other administrative districts within the country. Even though our survival curve for the low‐risk group patients resembles that of the developed countries, a large proportion of our patients were diagnosed with higher stage and clinical risk group. This study highlights the need for a nationwide collaborative practice to improve the early diagnosis and initiation of therapy for patients with RMS.

Another area of concern we wanted to address was the access to care for patients across the continuum, particularly in terms of avoiding gender bias. We report a male to female ratio of 2.4:1, a gender distribution which is comparable to international figures, thus supporting the similar provision of healthcare services for males and females. For the purposes of this report, gender disparity as a factor in disease influence and outcome was not seen in our patient mix.

While the incidence of the different subtypes of RMS in our population was not different from the internationally reported data (78% ERMS, 19% ARMS and 3% spindle), an additional limitation identified by our study is the low rate of cytogenetic and molecular testing which is increasingly being used as part of diagnostic evaluation and risk stratification as well as to predict clinical outcome of the disease. Historically, rhabdomyosarcoma at our center has been more of a morphological diagnosis with further validation of diagnosis and or subtype by using immunohistochemical stains such as Desmin, Myogenin, and myoD1. Cytogenetics has only been used to confirm alveolar subtype when the morphology was inconclusive. We found that only 44 patients were tested for the FOXO1 fusion gene using FISH. The results confirmed alveolar RMS in 8 of the 44 patients or 18.2%. Interestingly, out of the remaining 36 patients who were negative for the FOXO1 gene fusion, 21 showed chromosomal aberrations (extra copies of 13q in 16 patients; loss of 13q in 2 cases; and 18q loss, tetrasomy 2 in one patient, and pentasomy 8 in one patient). Though chromosomal deletions and gains are more characteristic of embryonal RMS with frequent occurrence of 11p15 loss, the clinical significance of our findings is not clear.^3^ Efforts should be made to incorporate cytogenetic and molecular techniques in our clinical practice and research work.

Lastly, comparing the survival data between the local hybrid institutional protocols used initially at our center and the COG regimens applied at a later stage across the different risk groups showed no statistically significant difference. The relatively shorter follow up period for the latter group makes it difficult to reach a strong conclusion when comparing the different treatments.

## 5 CONCLUSION

This retrospective analysis has been enlightening in that we have been able to address, assess, and evaluate several areas across the continuum of management for RMS in the pediatric population. Several important areas have been identified as focus points moving forward in our management of this disease. Additionally, the need for a national collaborative effort with uniform, standardized care is much needed to support best outcomes for this patient population.

## AUTHOR CONTRIBUTIONS

All authors had full access to the data in the study and take responsibility for the integrity of the data and the accuracy of the data analysis. Conceptualization, F.M.L.; Methodology, F.M.L.; Investigation, F.M.L.; Formal Analysis, F.M.L.; Resources Writing, F.M.L.; Original Draft, F.M.L.; Writing ‐ Review & Editing, F.M.L.; Visualization, F.M.L.; Supervision, F.M.L.; Funding Acquisition, F.M.L.

## CONFLICT OF INTEREST

None of the authors has any affiliation that could be considered relevant and important with any organization that has a direct interest, particularly a financial interest, in the subject matter discussed.

## Data Availability

The data that support the findings of this study are available from the corresponding author upon reasonable request.
